# Leveraging Dissolution by Autoinjector Designs

**DOI:** 10.3390/pharmaceutics14112544

**Published:** 2022-11-21

**Authors:** Christoph Spangardt, Christoph Keßler, Ramona Dobrzewski, Antonia Tepler, Simon Hanio, Bernd Klaubert, Lorenz Meinel

**Affiliations:** 1 Institute of Pharmacy and Food Chemistry, University of Wuerzburg, Am Hubland, 97074 Wuerzburg, Germany; 2 Central Institute of the Bundeswehr Medical Service Munich, Ingolstaedter Landstraße 102, 85748 Garching, Germany; 3 Helmholtz Institute for RNA-Based Infection Research (HIRI), Josef-Schneider-Strasse 2, 97080 Wuerzburg, Germany

**Keywords:** autoinjector, dissolution, oxime, response surface, nerve agent

## Abstract

Chemical warfare or terrorism attacks with organophosphates may place intoxicated subjects under immediate life-threatening and psychologically demanding conditions. Antidotes, such as the oxime HI-6, which must be formulated as a powder for reconstitution reflecting the molecule’s light sensitivity and instability in aqueous solutions, dramatically improve recovery—but only if used soon after exposure. Muscle tremors, anxiety, and loss of consciousness after exposure jeopardize proper administration, translating into demanding specifications for the dissolution of HI-6. Reflecting the patients’ catastrophic situation and anticipated desire to react immediately to chemical weapon exposure, the dissolution should be completed within ten seconds. We are developing multi-dose and single-dose autoinjectors to reliably meet these dissolution requirements. The temporal and spatial course of dissolution within the various autoinjector designs was profiled colorimetrically. Based on these colorimetric insights with model dyes, we developed experimental setups integrating online conductometry to push experiments toward the relevant molecule, HI-6. The resulting blueprints for autoinjector designs integrated small-scale rotor systems, boosting dissolution across a wide range of viscosities, and meeting the required dissolution specifications driven by the use of these drug products in extreme situations.

## 1. Introduction

The exposition of chemical weapons puts the time factor at the center of all therapeutic efforts. A prime example of chemical warfare agents used in recent years is the class of organophosphates (OP). High-profile cases include but are not limited to the attempted assassinations of Sergei and Julia Skripal [1] as well as the attack on Alexei Navalny [2]. The 1995 attack on the Tokyo subway resulted in 640 victims [3], and the sad use of chemical weapons in Syria comes to mind [4]. Prime examples of OP compounds are tabun (GA), sarin (GB), soman (GD), VX, and the more recently developed novichok class of agents. Published data, especially on the latter, is scarce, yet its use in the last decade is a reason for general concern due to its high lethality [5].

OP absorption after an attack is primarily dermal and by inhalation [6]. OP exposure leads to the phosphorylation of serin within the active center of acetylcholinesterase (AChE) [7]. In the second step referred to as aging, dealkylation occurs, irreversibly inactivating the enzyme. For example, aging half times of soman-inhibited AChE are in the range of  ~ 2 min [8]. Oxime therapy must occur before aging takes place to maximize clinical success. These facts trigger challenging pharmaceutical specifications on drug products. For example, HI-6, an oxime valuable for its rapid AChE reactivation, has unfavorable physico-chemical properties driving the need for complex development efforts. It is unstable when exposed to UV light [9] and rapidly degrades in aqueous solutions [10,11,12,13]. Consequently, HI-6 must be light protected and formulated as a powder for reconstitution. This can be accomplished by continuous infusion for hospitalized patients or in mission/terroristic events with two chamber autoinjectors [14,15]. Complete dissolution within these autoinjectors must occur very rapidly. This demand reflects the pharmacological reasons mentioned above and the fact that the exposed will only have a short period of time before the symptoms take effect. At this point, handling, injection, or proper thinking become increasingly difficult due to anxiety and muscle tremors—this is why complete dissolution must be achieved quickly within common application times of auto-injectors, e.g., ten seconds [16]. Unfortunately, neither the HI-6 chloride salt nor alternative salts, such as HI-6 mesylate, meet dissolution requirements, which prevents timely dissolution and ultimately jeopardizes successful injection by the exposed [17]. Manual shaking improved the HI-6 dissolution of both salts but not to an extent reaching the ten-second window. These real-life experiences led to the specification of the 10-s window (activation to injection) reflecting the (anticipated) desire of an exposed person to react immediately to chemical weapon exposure, and it reflects the (anticipated) inability of these patients to wait (e.g., for the completion of the dissolution processes) in light of (anticipated) high stress, perhaps panic, in these catastrophic situations [18].

Lastly, HI-6 dissolution is temperature-dependent [19]—taken together, quite challenging physico-chemical compound properties meet extremely challenging pharmaceutical specifications reflecting the time course and severity of the symptoms following attacks. A further complication is that antidotal treatment autoinjectors may contain atropine and an anxiolytic to achieve a sufficient treatment [20]. Therefore, the autoinjectors’ successes demand shaking after activation (Astra Tech/STI design). Interestingly, the autoinjector designs guide the solvent through filters directly into the powder bed (Meridian Medical Technologies) [19,20,21]. However, proper shaking under chemical attack is a challenge (*vide supra*; Astra Tech/STI design), and incomplete dissolution is a challenge for both designs and perhaps more strongly for the one using filters, which may be blocked by undissolved drug substance. Generally, autoinjectors for military or homeland security use may be used in exceptional situations [22]. Examples are morphine autoinjectors on the battlefield [23,24], antidotes (*vide supra*) [25], or emergency anaphylactic treatments [26]. In the case of the Bundeswehr, several autoinjectors have been stockpiled for many years, triggering the need for long-term stability [27].

This study presents the development of muti-dose and single-dose autoinjectors meeting the still unmet specification—HI-6 dissolution within less than ten seconds.

## 2. Materials and Methods

### 2.1. Materials

HI-6 dimethyl sulfonate (DMS) was a kind donation from Ferak GmbH (Berlin, Germany), while HI-6 dichloride (Cl_2_) was manufactured by Astra (Sodertalje, Sweden). Sodium hydrogen phosphate dihydrate and atropine sulfate monohydrate were purchased by Merck KGaA (Darmstadt, Germany). Anhydrous glycerol was acquired by Sigma-Aldrich (St. Louis, MO, USA). Carboxymethylcellulose sodium (sodium CMC) was bought from JRS Pharma GmbH (Rosenberg, Germany). Lugol’s solution was bought off the shelf from Sigma-Aldrich (St. Louis, MO, USA) as was sodium thiosulfate. Deionized purified water (Millipore water, hereafter referred to as water) was generated by an in-house Millipore purification system from Merck KGaA. All other standard chemicals and laboratory consumables, if not stated otherwise, were purchased from either VWR International GmbH (Ismaning, Germany) or Sigma-Aldrich.

### 2.2. Methods

#### 2.2.1. Colorimetry

**Figure 1:**
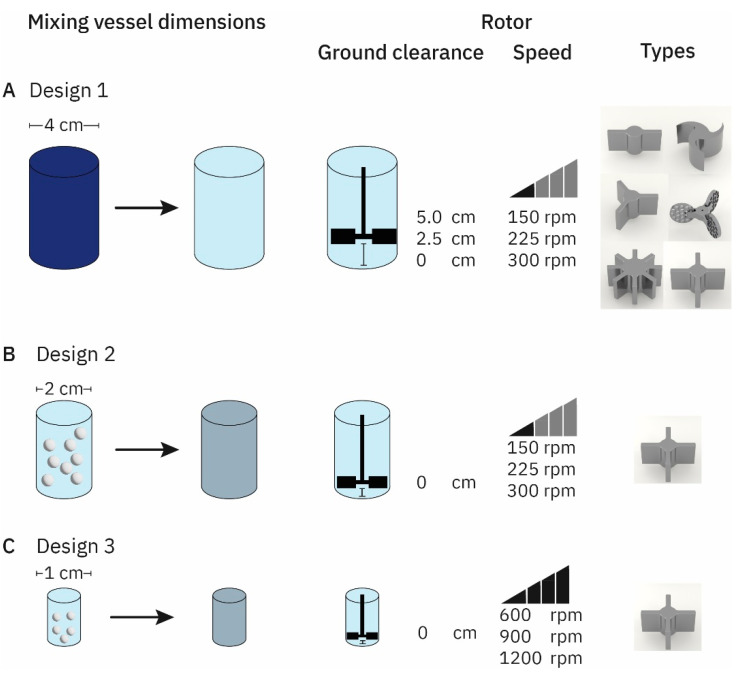
(**A**) Setup for the discoloration assay using a mixing vessel with an internal diameter of 4 cm. The stirrer operated at a ground clearance levels between 0 cm and 5 cm and at a rotational speed of 150 to 300 rpm. Six different types of rotors were compared. (**B**) Conductometric setup for a multi-dose autoinjector surrogate. The four-blade rotor geometry was selected for these designs. The stirrer operated at the bottom of the t mixing vessel at rotational speed ranging between 150 rpm and 300 rpm. (**C**) Conductometric setup for a single-dose autoinjector with an internal diameter of 1 cm. The four-blade rotor was positioned at the bottom and was operated at rotational speed between 600 rpm and 1200 rpm.

**Figure 2:**
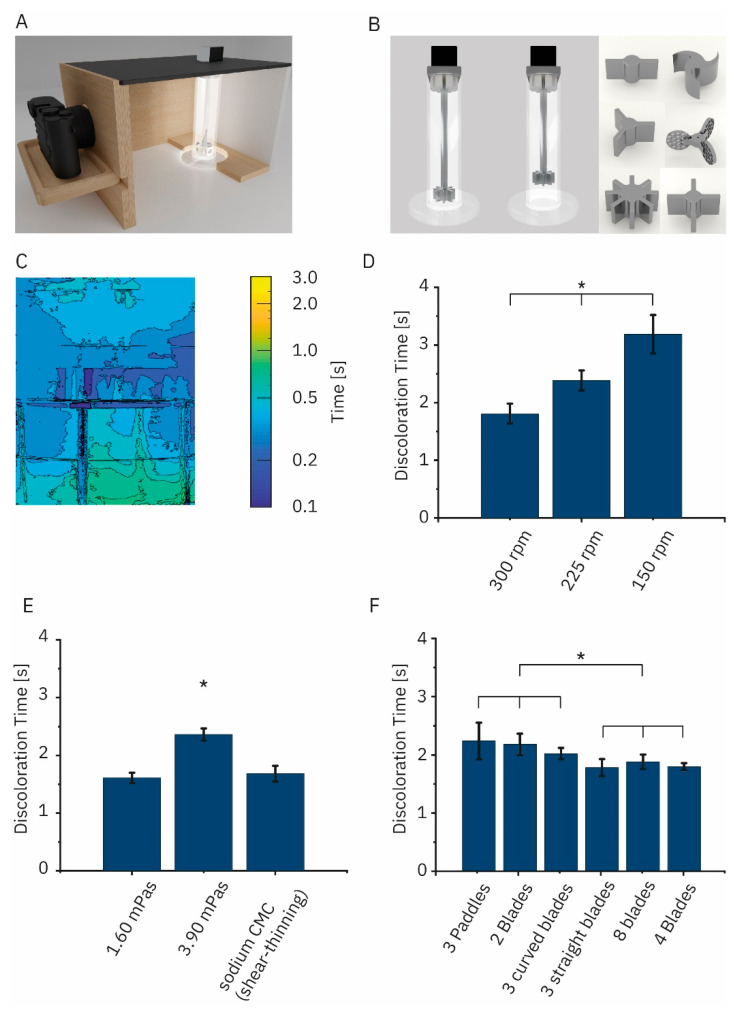
(**A**) Setup for the discoloration time measurement of Lugol’s solution using sodium thiosulfate solution, consisting of a mixing vessel with a stepper motor, an illuminated box with a camera mount, as well as an HD-capable camera (this is introduced in Figure 1A). (**B**) Detailed view of the mixing vessel with a rotor in the bottom position (**left**) and positioned at a ground clearance of 5 cm (**middle**). Furthermore, the six different rotor geometries evaluated are depicted (**right**). (**C**) Time until a pixel was first determined as mixed by the analysis algorithm using an eight-blade mixer rotating at 300 rpm at a ground clearance of 5 cm in water. (**D**) Discoloration time for an eight-blade mixer with different rotational speeds in water (0.89 mPa s). (**E**) Discoloration time for an eight-blade mixer in media with increasing viscosity (Newtonian) or shear-thinning (carboxymethylcellulose sodium (sodium CMC) properties at 300 rpm (we cannot report a viscosity for sodium CMC. CMC is shear thinning. The vessel has a gradient of different shear stresses. For the CMC’s shear-thinning properties, the gradient translates into a range of viscosities within the solution which was not experimentally accessible (mean ± standard deviation; * *p* < 0.05, ANOVA with Tukey post hoc test; *n* = 5). (**F**) Discoloration times in water at a ground clearance of 5 cm and a rotational speed of 300 rpm for the different rotors depicted in panel (**B**) (mean ± standard deviation; * *p* < 0.05, ANOVA with Tukey post hoc test; *n* = 5).

Different vessel dimensions were tested (Figure 1). The setup for the discoloration assay was manufactured by the institute’s workshop and consisted of a polymethylmethacrylate (PMMA) mixing vessel with a 4 cm diameter that was locked in place inside a wooden box (Figure 2A). The wooden box itself consisted of a shelf holding the camera system used to record the discoloration videos, lighting, stepper motor housing, and a point to fix the mixing vessel. Constant lighting was achieved by installing a LED ring light above the mixing vessel. Videos were taken on a Leica Q (focal length 28 mm, aperture: f/8; Leica camera AG, Wetzlar, Germany) in HD (1920 × 1080 pixels) at a frame rate of 60 s^−1^. The mixers were powered using a stepper motor (JKonMotor, Changzhou, China) that was controlled using custom programming on an Arduino uno (Arduino, Sommerville, MA, USA) platform. Programming allowed for three different rotor speeds (150 rpm, 225 rpm, and 300 rpm) and three presets of revolutions (10, 15, and 20). All combinations could be accessed using a keypad (for a comprehensive overview see Figure 1A).

All mixers were designed and constructed in the institute’s workshop from aluminum on a Datron neo-CNC milling machine (Datron AG, Muehltal, Germany).

For each experiment, 100 mL of water and 20 mL of starch solution (β = 1% [w v^−1^]) were colored using 40 µL of lugol’s solution. To discolor the assay, 40 µL of a sodium thiosulfate solution (c = 1.0 mol L^−1^) was added. Simultaneously, the rotor was started and the whole process was filmed.

The videos were manually processed using Adobe Photoshop 23 (Adobe Inc., San José, CA, USA) and analysis was performed using a custom MATLAB algorithm (Matlab R2019b, The Mathworks Inc., Natick, MA, USA). The algorithm was programmed based on the work of Vega-Alvarado et al. [28] and Cabaret et al. [29]. In all experiments the medium changed color from deep blue to colorless. Image processing was necessary as the camera captures a wider field beyond the dimensions of the mixing vessel. Therefore, we cropped the images of the video to measure only the region of interest (ROI); mixing container and content but not the environment outside of the container.

The algorithm then converted all images to grayscale ones, assigning each pixel a value from 0 to 255. Furthermore, each pixel was assigned a x and y coordinate in a two-dimensional projection of the mixing vessel. Then, the first and last frames of each video were used to calculate the individual threshold value, $\beta_{x,y}^{\mathrm{gray}}$. Each frame then was treated as a matrix and assigned an index value ranging from k=1 to k=n, where n was the total number of frames in each video. Based on this, $\beta_{x,y}^{\mathrm{gray}}$ was defined by Equation (1).

(1) $$\beta_{x,y}^{\mathrm{gray}}=R_{x,y}^{0}+X\;(R_{x,y}^{\infty }-R_{x,y}^{0})$$

The value, $\beta^{\mathrm{gray}}$, was assigned to every pixel with the coordinates, x, y, from the reference pixel, R, at t = 0 and t = ∞ with a fixed mixing coefficient, X = 0.5, as suggested before [29] and in good agreement with naked eye observation of the color change from blue to colorless. From this, in each frame every pixel was compared with its individual reference value. A pixel is categorized as mixed whenever $R_{x,y}\left(t\right)>{\;\beta }_{x,y}^{\mathrm{gray}}$. This operation was performed for every pixel in every frame. The result was saved as a logical output where $R_{x,y}\left(t\right)\geq {\;\beta }_{x,y}^{\mathrm{gray}}=1$ and $R_{x,y}\left(t\right)<{\;\beta }_{x,y}^{\mathrm{gray}}=0$. Subsequently, the ratio of pixels mixed (M) was computed according to Equation (2).

(2) $$M=\frac{n\;\left(\mathrm{mixed}\;\mathrm{pixels}\right)}{n\;\left(\mathrm{total}\;\mathrm{number}\;\mathrm{of}\;\mathrm{pixels}\right)}\;*\;100$$

The discoloration times was defined as the time, t, when M reached 90%. All experiments assessing autoinjector activation were performed in quintuplicate at standard ambient temperature (25 °C).

#### 2.2.2. Conductometry

Conductometric assays were performed in two differently sized mixing vessels with 2 cm (Figure 1B) and 1 cm (Figure 1C) internal diameter. They were made from PMMA in the in-house workshop. Online conductometric data were recorded using the conduino system developed by Carminati et al. [30]. This system consists of an Arduino uno that serves as a control unit as well as a custom printed circuit board (PCB) allowing for up to four electrodes to be measured simultaneously. Due to the mixing vessels’ sizes, a single electrode setup was chosen. Furthermore, a powder drop off was constructed. A leaf shutter as well as a vibration engine controlled the addition of the powder to the mixing vessel and minimized powder residue. USB 2.0 cables (Hama GmbH, Monheim, Germany) were prepared according to the conduino 1.2 protocol [31]. To exclude the influence of the experimenter on the results, the stepper motor, data acquisition, and powder drop off were synchronized. Files were recorded and data was analyzed using the above-mentioned MATLAB-algorithm.

To compensate for inherent differences in conductivity readout, all graphs then were processed using Origin 2020 (Originlab Inc., Northampton, MA, USA). Processing consisted of standardizing the readout on a per-run basis (0% to 100%). Dissolution time was set to the 90th percentile of the maximum readout. All assays were performed in triplicate.

In the larger sized vessel, also dubbed as multi-dose, 10 mL of solvent was added, whereas only 3 mL of liquid was added in the smaller sized one (also denoted as single-dose). Solvents were prepared according to Table 1.

The particle size distributions of both HI-6 salts were determined under the microscope. If not otherwise noted, all experiments were performed at standard ambient temperature (25 °C).

## 3. Results

### 3.1. Colorimetric Analysis of Sodium Thiosulfate Discoloration

We analyzed the discoloration of starch-Lugol’s solution with sodium thiosulfate solution in a stirred vessel by capturing an HD-video file of the redox reaction (Figure 1A). Resulting outcomes fed an algorithm classifying each pixel as mixed or unmixed in the region of interest on a frame-by-frame basis. This analysis was performed for the six rotor geometries (Figure 2B). We visualized the time until a pixel was first determined as mixed in contour plots (Figure 2C). Different factors influenced the discoloration time. The rotational speed of the mixer was inversely proportional to the discoloration time (Figure 2D). In the case of an eight-blade mixer, discoloration times increased from 1.88 ± 0.12 s to 2.38 ± 0.17 s and 3.19 ± 0.17 s for 300 rpm, 225 rpm, and 150 rpm, respectively. Dynamic viscosity did not always impact discoloration times. An increase in viscosity from 0.89 mPa s (water) to 1.60 mPa s (22.5% glycerol solution) had no statistically significant impact on the discoloration time (1.72 ± 0.17 s and 1.61 ± 0.09 s, respectively). A viscosity of 3.90 mPa s (45% glycerol solution) significantly increased the discoloration time to 2.36 ± 0.10 s. Discoloration times in a shear-thinning 0.5% sodium CMC solution did not significantly change either (1.68 ± 0.14 s) (Figure 2E). The rotor geometry impacted the discoloration as well (Figure 2F). Discoloration times ranged from 1.78 ± 0.15 s for a three-blade rotor over 1.79 ± 0.06 s (four-blade rotor) and 1.88 ± 0.12 s (eight blades) to values of 2.03 ± 0.10 s (three curved blades) and 2.18 ± 0.18 s (two blades). The longest discoloration time was measured at 2.24 ± 0.31 s (three paddles). Sodium dihydrogen phosphate was ‘freely soluble’ in all tested media according to the classification of the European Pharmacopoeia (Ph. Eur 10.0/5.11).

### 3.2. Solid Drug Dissolution in Autoinjectors

#### 3.2.1. Factors Impacting Powder Dissolution

**Figure 3:**
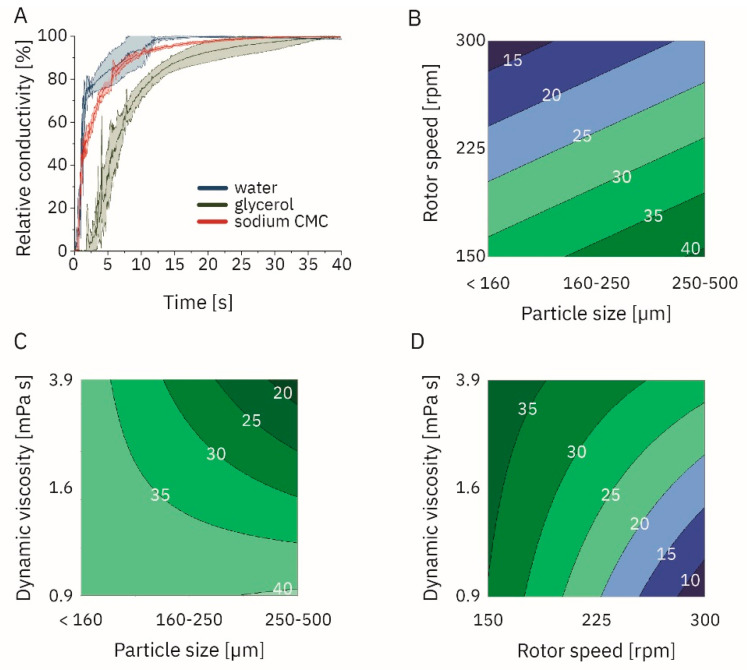
(**A**) Time series of the relative conductivity for the dissolution of monobasic sodium hydrogen phosphate dihydrate in a mixing vessel the size of a multi-dose autoinjector at 300 rpm and particle size <160 µm (this is introduced in Figure 1B). Effects of (**B**) rotor speed and particle size, (**C**) dynamic viscosity and particle size, (**D**) dynamic viscosity and rotor speed on the dissolution time as indicated by white numbers and in s.

Rotor speed, dynamic viscosity of the Newtonian solvent, and particle size significantly impacted the dissolution time (α = 0.05; Tables S1 and S2; Figure 1B and Figure 3). In contrast, there was significant influence of different temperature levels (0, 25, and 45 °C) on dissolution times. The analysis assessed the mixing of sodium hydrogen phosphate by conductometry. Furthermore, the significant interaction was between ‘particle size—dynamic viscosity’ and ‘rotor speed—dynamic viscosity’. Dissolution times ranged from 6.31 s (particle size <160 µm, rotor speed 300 rpm, dynamic viscosity 0.89 mPa s, temperature 45 °C) to 51.18 s (particle size 250 µm to 500 µm, rotor speed 150 rpm, dynamic viscosity 3.90 mPa s, temperature 5 °C).

We further profiled shear-thinning solutions using sodium CMC compared to water and Newtonian glycerol solutions (Table S3; Figure 3). The dissolution times ranged from 3.91 ± 0.45 s (particle size < 160 µm, rotor speed 300 rpm, temperature 25 °C) to 34.62 ± 1.78 s (particle size < 160 µm, rotor speed 150 rpm, temperature 25 °C).

#### 3.2.2. Dissolution of HI-6 in the Active Autoinjectors

**Figure 4:**
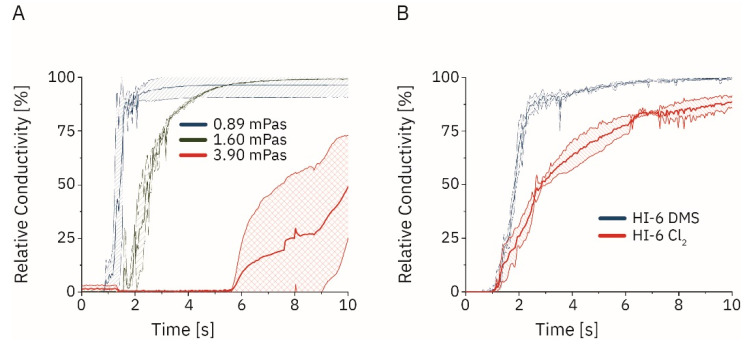
(**A**) Dissolution of sodium dihydrogen phosphate dihydrate in water and glycerol solutions with increasing dynamic viscosities (*n* = 3; introduced in Figure 1C). (**B**) Dissolution of HI-6 dimethyl sulfonate (DMS) and HI-6 dichloride (Cl_2_, *n* = 3; T = 25 °C) in aqueous solutions containing 0.33 mg mL^−1^ atropine.

We now expanded the insights reported above for multi-dose to single-dose active autoinjectors (the following was introduced in Figure 1C). The dissolution of different compounds was recorded using a self-engineered online conductivity probe and the resulting time series was processed using Originlab. The impact of viscosity and the stirring speed was initially studied with sodium hydrogen phosphate (particle sizes <160 µm; Figure 4A). Dissolution times of 1.96 ± 0.10 s (at 600 rpm), 2.32 ± 1.01 s (900 rpm), and 1.50 ± 0.22 s (1200 rpm) were observed in water. Increasing the viscosity to 1.60 mPa s increased the dissolution times to 4.39 ± 0.35 s (600 rpm), 3.22 ± 0.13 s (900 rpm), and 3.91 ± 0.10 s (1200 rpm). Dynamic viscosities of 3.90 mPa s prevented complete salt dissolution within the experimental time window of 60 s at 600 rpm. Higher rotation speeds reduced the dissolution times to 27.40 ± 4.56 s (900 rpm) and 18.47 ± 0.96 s (1200 rpm). Dissolution experiments were expanded to HI-6 (Cl_2_) (mean particle size 178 ± 66 µm; Figure S1**)** and HI-6 DMS (mean particle size 128 ± 51 µm) in 3 mL solution of water and in an aqueous solution with 0.33 mg mL^−1^ atropine sulfate (Figure 4B). These particle sizes were similar to the ones used in previous studies [32]. Dissolution time, set as the 90th percentile of the maximum conductivity on a per-run basis, at 1200 rpm using a four-blade stirrer was significantly different for the dichloride and the DMS salt with 9.13 ± 1.15 s and 1.50 ± 0.44 s, respectively (* *p* < 0.05, Student’s *t*-test; *n* = 3).

## 4. Discussion

We developed blueprints for multi-dose and single-dose active autoinjectors. These autoinjectors provided complete dissolution of therapeutically relevant HI-6 concentrations within less than two seconds, thereby addressing an unmet pharmaceutical need in military and emergency pharmacy and medicine.

We selected moderate top rotor speeds, despite previously published direct correlations of rotor speed and dissolution times [33]. However, these desirable higher rotor speeds are not easy to meet in our setting as we wanted to keep comparable designs throughout the scale-up. For example, the experimental designs outlined in Figure 1A,C had four-fold changes in the inner diameter of the autoinjector (Figure 1). In order to keep the dimensionless Reynolds number constant, which we used for upscaling, a 16-fold increase in rotor speed would be required [34]. The resulting power needs of the rotor for the larger designs would challenge the technical translation of future carry-on autoinjector designs [35].

We started with a colorimetric analysis of the flow patterns, similar to previous reports [29,36,37,38]. However, the conductometry setup has, to our knowledge, not been used for the measurement of dissolution. One limitation of this setup is the different baseline conductivity values in different solvents. Therefore, comparing absolute conductivity values might be misleading. Consequently, values were normalized in our experiments, and relative conductivities were reported. Compared to the colorimetric approach, the advantage is the independence from chromophores and a broad application. Furthermore, we were able to determine the dynamic viscosity as a significant factor (Figure 3C,D) which we were not able to in every case in the colorimetric approach, indicating a higher discriminatory power of the conductometric setup. The result is in agreement with previously published data (*vide supra*) [39,40]. Sodium dihydrogen phosphate and HI-6 DMS dissolution was under sink conditions according to European Pharmacopeia (Ph. Eur. 10.0/5.1.7), while the dissolution of HI-6 Cl_2_ was not, posing a contributing factor to the longer dissolution times [41]. We observed a larger mean particle size for HI-6 Cl_2_ compared to HI-6 DMS that was smaller than previously reported [32] and also contributed to the longer dissolution times.

This study provides blueprints for future autoinjectors serving extreme needs after chemical attacks. This technical approach also leads to much shorter application times compared to marketed products, which is important in mass casualty events. However, the blueprints are not final, and several future development steps are required before a final product becomes available. For example, the rotor shaft may be further miniaturized. Furthermore, the powder should be stored in a separate compartment within the autoinjector. The power source, motor, etc., must be integrated into the autoinjector design. The stepper motor used in our experiments was designed to precisely control the experimental conditions and is not ideal for a marketed product. Designs for marketed products may allow even faster rotor speeds and must be robust under harsh environmental conditions (e.g., extreme climate zones and combat conditions). Whether this may be achieved using a spring powered or an electrically powered rotor is the subject of future experiments, especially keeping in mind the limitations regarding the device’s size and weight.

The findings reported here may be helpful for other technological applications, for example the injection of viscous fluids [42,43,44,45]. Despite their small size and energy introduced by the rotors, the systems reported here introduced high shear forces. These shear forces may be valuable for shear-thinning solutions, which despite their high baseline viscosity can be injected after shearing. One future application may be for calcium phosphate pastes used in dentistry [46]. Other applications may be highly concentrated antibody formulations (up to some grams per milliliter) that use rotor-induced shear thinning to reduce the ejection force. Thereby, these highly viscous formulations may become injectable with a reasonable force of e.g., about 80 N [47].

## Figures and Tables

**Table 1:** Liquid media used in the dissolution setup.

Medium	Dynamic Viscosity [mPa s]
water	0.89
22.5% glycerol	1.60
45.0% glycerol	3.90
0.5% carboxymethylcellulose sodium	18,000−3.6 ^1^

^1^ Shear thinning medium viscosity recorded between shear rates of 2 s^−1^ to 10,000 s^−1.^

## Data Availability

Data sets supporting the reported results are made available via the authors on request.

## Supplementary Materials

The following supporting information can be downloaded at: https://www.mdpi.com/article/10.3390/pharmaceutics14112544/s1, Table S1: Conditions of multi-dose dissolution experiments and their respective dissolution times; Table S2: Analysis of variance of the significant factors with regard to dissolution time; Table S3: Conditions of multi-dose dissolution experiments in sodium CMC at 25 °C and their respective dissolution times; Figure S1: Histogram for the particle size distribution of (A) HI-6 dimethyl sulfonate (DMS) and (B) HI-6 dichloride (Cl_2_) with corresponding microscopy images for (C) DMS and (D) Cl_2_.
