# Supplementary material for: Leveraging Dissolution by Autoinjector Designs
Source: Pharmaceutics. 2022 Nov 21;14(11):2544. doi: 10.3390/pharmaceutics14112544 (PMC9695427; doi:10.3390/pharmaceutics14112544)
Supplement: Supplementary file 1 [file pharmaceutics-14-02544-s001.zip › pharmaceutics-2019890-supplementary.pdf]

Table S1. Conditions of multi-dose dissolution experiments and their respective dissolution times (given as mean ± standard deviation; *n* = 3).

Particle Size [µm]	Rotor Speed [rpm}	Solvent	Temperature [°C]	Dissolution Time [s]
<160	150	Water	5	38.31 (±4.77)
<160	150	Water	45	40.18 (±0.56)
<160	150	45% Glycerol	5	22.20 (±2.53)
<160	150	45% Glycerol	45	41.40 (±6.75)
<160	300	Water	5	7.01 (±2.20)
<160	300	Water	45	6.31 (±2.56)
<160	300	45% Glycerol	5	18.88 (±1.87)
<160	300	45% Glycerol	45	14.87 (±4.36)
250–500	150	Water	5	26.74 (±3.16)
250–500	150	Water	45	37.51 (±7.75)
250–500	150	45% Glycerol	5	51.18 (±1.89)
250–500	150	45% Glycerol	45	44.71 (±2.21)
250–500	300	Water	5	10.90 (±2.70)
250–500	300	Water	45	8.12 (±0.70)
250–500	300	45% Glycerol	5	44.88 (±2.89)
250–500	300	45% Glycerol	45	35.26 (±1.10)
<160	225	25% Glycerol	25	25.01 (±2.68)
250–500	225	25% Glycerol	25	29.78 (±0.55)
160–250	150	25% Glycerol	25	26.58 (±13.96)
160–250	300	25% Glycerol	25	8.24 (±0.26)
160–250	225	Water	25	16.93 (±5.29)
160–250	225	45% Glycerol	25	37.94 (±2.25)
160–250	225	25% Glycerol	5	36.57 (±5.78)
160–250	225	25% Glycerol	45	24.45 (±7.74)
160–250	225	25% Glycerol	25	25.23 (±7.74)

Table S2. Analysis of Variance of the significant factors with regard to dissolution time.

Source	DF	Seq SS	Contribution [%]	Adj. SS	F-Value
Model	5	10683.8	82.62	10683.8	61.79
**Linear**	3	8231.5	63.65	7981.6	76.94
Particle Size	1	1121.0	8.67	1066.9	30.85
Rotor Speed	1	5087.6	39.34	4992.7	144.38
Glycerol	1	2022.8	15.64	1889.8	54.65
**2-way Interaction**	2	2452.3	18.96	2452.3	35.46
Particle Size * Glycerol	1	1535.5	11.87	1589.6	45.97
Rotor Speed * Glycerol	1	916.8	7.09	916.8	26.51
Error	65	2247.7	17.38	2247.7	
Total	70	12931.5	100.0		

Table S3. Conditions of multi-dose dissolution experiments in sodium CMC at 25 °C and their respective dissolution times (given as mean ± standard deviation; *n* = 3).

Particle size [µm]	Rotor Speed [rpm}	Dissolution time [s]
<160	150	34.62 (±1.78)
<160	225	33.23 (±3.77)
<160	300	3.91 (±0.45)
160–250	150	34.16 (±0.10)
160–250	225	23.77 (±1.90)

160–250	300	7.21 (±0.84)
250–500	150	29.08 (±1.42)
250–500	225	26.50 (±3.86)
250–500	300	13.25 (±0.24)

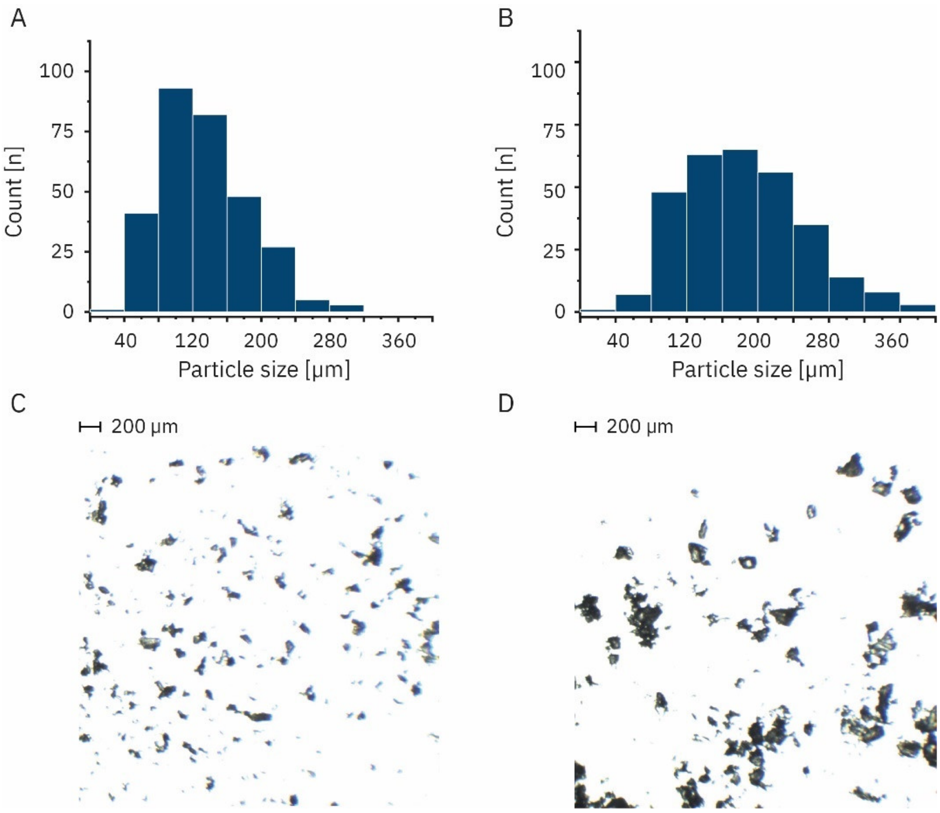

Figure S1. Histogram for the particle size distribution of (**A**) HI-6 dimethyl sulfonate (DMS) and (**B**) HI-6 dichloride (Cl_2) with corresponding microscopy images for (**C**) DMS and (**D**) Cl_2. Microscope images were recorded with a Bresser microscope and a Bresser MicroCam (5.0MP) camera at a fourty times magnification. Particle sizes were determined with the MicroCamLab software version 7.3.1.8.
